# Elotuzumab Enhances CD16-Independent NK Cell-Mediated Cytotoxicity against Myeloma Cells by Upregulating Several NK Cell-Enhancing Genes

**DOI:** 10.1155/2024/1429879

**Published:** 2024-02-27

**Authors:** Yan-Hua Wang, Shotaro Hagiwara, Hiroshi Kazama, Yuki Iizuka, Norina Tanaka, Junji Tanaka

**Affiliations:** ^1^Department of Hematology, Tokyo Women's Medical University, 8-1, Kawada-Cho, Shinjuku-Ku, Tokyo 162-8666, Japan; ^2^Department of Medicine, Tokyo Women's Medical University, Adachi Medical Center, 4-33-1, Kohoku, Adachi-Ku, Tokyo 123-8558, Japan

## Abstract

Multiple myeloma (MM) is an intractable hematological malignancy caused by abnormalities in plasma cells. Combination therapy using antibodies and natural killer (NK) effectors, which are innate immune cells with safe and potent antitumor activity, is a promising approach for cancer immunotherapy and can enhance antitumor effects. Elotuzumab (Elo) is an immune-stimulatory antibody that targets the signaling lymphocytic activation molecule family 7 (SLAMF7) expressed on the surface of MM and NK cells. We confirmed that Elo strongly promoted NK cell-mediated antibody-dependent cellular cytotoxicity (ADCC) against SLAMF7-positive MM cells in a CD16-dependent NK cell line, and also activated expanded NK cells derived from peripheral blood mononuclear cells of healthy donors and patients with MM in the present study. However, the antitumor effects and genes involved in the direct promotion of NK cell-mediated activation using Elo in CD16-independent NK cells are not clearly known. In this study, we demonstrated that Elo pretreatment significantly enhanced CD16-independent NK cell-mediated cytotoxicity in both SLAMF7-positive MM.1S and SLAMF7-negative K562, U266, and RPMI 8226 tumor cells. Upon direct simulation of CD16-independent NK cells with Elo, increased levels of CD107a degranulation and IFN-*γ* secretion were observed along with the upregulation of granzyme B, TNF-*α*, and IL-1*α* gene expression. The enhanced NK cell function could also be attributed to the increased expression of the transcription factors T-BET and EOMES. Furthermore, the augmentation of the antitumor effects of CD16-independent NK cells upon pretreatment with Elo enhanced the expression of CRTAM, TNFRSF9, EAT-2, and FOXP3 genes and reduced the expression of HSPA6. Our results suggest that Elo directly promotes the cytotoxic function of CD16-independent NK cells against target cells, which is associated with the upregulation of the expression of several NK cell-enhancing genes.

## 1. Introduction

Multiple myeloma (MM) is the second most common hematologic malignancy, caused by the abnormal proliferation of plasma cells in the bone marrow [1]. Over the past decade, the application of novel therapeutics, including proteasome inhibitors (such as bortezomib), immunomodulatory drugs (such as lenalidomide and pomalidomide), and immunotherapy agents targeting MM-associated antigens has delayed disease progression and improved the overall survival of patients with MM [2]. However, MM still remains an intractable disease because of relapse and is refractory to therapies in almost all patients. In addition, the presence of minimal residual disease affects outcomes [3, 4]. Natural killer (NK) cell activity is decreased or exhausted depending on the clinical stage of MM in patients [5]. Several factors are possibly involved in myeloma cells escaping immunity, including the overexpression of programed cell death protein 1 (PD-1) ligand, recruitment of T regulatory cells (Tregs) [6], and the complex genetic heterogeneity of MM itself [1, 2]. Thus, there is a need for diversified therapeutic strategies for patients with relapsed and/or refractory MM (RRMM). Enhancing or restoring the antitumor activity of innate immune cells using monoclonal antibodies (mAbs) that target myeloma-related antigens is an emerging therapeutic approach.

NK cells are key immune cells with a large granular lymphoid population (CD56^+^ and/or CD16^+^, CD3^−^) that have an innate ability to destroy tumor cells as effectors do not require the recognition of major histocompatibility complex or human leukocyte antigen (HLA) molecules [7]. NK cells can integrate with ligands on the surface of target cells through signaling receptors and induce cytokine production to increase cytotoxicity in tumor cells [8]. NK-mediated antitumor therapy is a safe immunotherapeutic method that plays an important role as a supplement to frontline cancer treatments [9]. In recent years, antibody therapy, in combination with NK cells, has been reported to enhance the antitumor effect of NK cells in cancer immunotherapy [10–12].

Elotuzumab (Elo) is one of the first antibodies approved for use in the combination treatment of patients with RRMM. It is a humanized IgG1 immune-stimulatory mAb against the extracellular domain of signaling lymphocytic activation molecule family 7 (SLAMF7, also known as CD319) [13]. SLAMF7, a receptor present on immune cells such as NK cells [14], is overexpressed on myeloma cells in patients with newly diagnosed MM and RRMM [13, 15]; thus, it is considered to be involved in the pathogenesis and progression of MM. Elo can be used to target SLAMF7 for anti-MM effects and has shown a low level of adverse events in clinical trials of combination therapies [16]. The antitumor effects of Elo are routed through the Fc receptor CD16-dependent NK cell-mediated antibody-dependent cellular cytotoxicity (ADCC) against SLAMF7-positive MM cells [14, 17]. We are currently conducting a Phase I/II clinical trial involving pretreatment with Elo–lenalidomide–dexamethasone in combination with *ex vivo* expansion and activated NK cell infusion therapy for patients with RRMM who remained minimal residual disease-positive after autologous hematopoietic stem cell transplantation [18]; we expect that it will augment the antimyeloma efficacy of NK cells *in vivo*. Moreover, Elo has been shown to directly promote NK cell-mediated cytotoxicity through Elo-SLAMF7 interaction without CD16 dependance in MM cells [19, 20]. Improvement in effector activity is also important for the combination treatment of patients with MM using Elo. However, not all patients have a sufficiently high number of CD16^+^ NK cells to respond to Elo *via* ADCC, and Elo stimulation is still insufficient to ensure the induction of NK cell-mediated cytotoxicity. Regarding the direct promotion of NK cell-mediated cytotoxicity using Elo without CD16 dependance, the mechanism and genes associated with this mode of activation are unclear, although a few studies have reported on this [19, 20].

In this study, we examined whether Elo can promote the ADCC effect of a CD16-positive NK cell line as well as that of human expanded autologous NK cells against target cells. Furthermore, we evaluated the antitumor effects of CD16-negative NK cells with and without Elo pretreatment on both SLAMF7-negative and SLAMF7-positive target cells. We also focused on identifying several genes associated with NK cell function in a CD16-independent manner after Elo stimulation.

## 2. Materials and Methods

### 2.1. Antibodies and Reagents

The anti-SLAMF7 antibody, Elo, with an Fc region was provided by Bristol Myers Squibb (New York, NY, USA). Purified isotype IgG1 mAb (#401402), anti-human PE-conjugated anti-CD319 (#331806) and anti-CD279 (PD-1, #367403), PE/Cy7-conjugated anti-CD16 (#302016) antibodies, and monensin solution (#420701) were purchased from BioLegend (San Diego, CA, USA). PE-conjugated anti-CD314 (also known as NKG2D, #A08934), CD3-FITC/CD(16 + 56)-PE (#A07735), and PE-Cy5-conjugated anti-CD56 (#A07789) were obtained from Beckman Coulter Life Sciences (Miami, FL, USA). FITC-conjugated anti-CD3 (#555332) and PE-Cy5-conjugated anti-CD107a (#555802) were obtained from BD Biosciences (San Jose, CA, USA). The recombinant human interleukin (IL)-2 (#170-076-148), IL-15 (#170-076-114), and MACS® GMP CD3 (OKT3, #170-076-124) were obtained from Miltenyi Biotec (Bergisch Gladbach, Germany). The CellTrace™ carboxyfluorescein diacetate succinimidyl ester (CFSE) Cell Proliferation Kit (#C34554, Invitrogen, Carlsbad, CA, USA) was used to stain target cells, while the Fixable Viability Dye eFluor® 660 (FVD, #65-0864, eBioscience, San Jose, CA, USA) was used to stain dead cells in the flow cytometry (FCM) analysis.

### 2.2. Cell Lines and Cell Culture

The human MM cell lines U266 (#TIB-196), MM.1S (#CRL-2974), and leukemia cell line K562 (#CCL-243) were purchased from the American Type Culture Collection (ATCC, Manassas, VA, USA), while another MM cell line RPMI 8226 (#IFO50013) was from the Japanese Collection of Research Bioresources (Osaka, Japan). These target (T) cells were cultured in complete medium RPMI-1640 (FUJIFILM, Osaka, Japan) supplemented with 10% fetal bovine serum (#10437-028, Gibco™, Thermo Fisher Scientific, Waltham, MA, USA) and 1% penicillin/streptomycin. The human NK-92MI cell line was obtained from ATCC (#CRL-2408), which has been shown to contain, express, and synthesize hIL-2; while NK-92MI/CD16a, a cell line exogenously transduced from the NK-92MI cells to express a 158V variant of high-affinity Fc*γ*RIIIa levels (CD16a), was kindly provided by Dr. Fumiaki Koizumi (Tokyo Metropolitan Komagome Hospital, Tokyo, Japan) [21, 22]. NK cell lines were grown in complete Minimum Essential Medium *α* (Invitrogen) containing 12.5% fetal bovine serum, 12.5% horse serum (#16050-122, Gibco™), and standard antibiotics. All cell lines were maintained in an incubator containing 5% CO_2_ at 37°C.

### 2.3. Expansion of Primary Human NK Cells

As an NK effector (E), we also used expanded primary NK cells (eNKs) from peripheral blood mononuclear cells (PBMCs) provided by three healthy donors and three patients with MM, collected from the Tokyo Women's Medical University Hospital (Tokyo, Japan). All procedures were conducted in accordance with the code of the Ethics Committee (approval no. 4479). The PBMCs were separated using Ficoll-Paque™ PLUS (Cytiva, Uppsala, Sweden) and cultured after checking NK cells by FCM, or immediately cryopreserved until use. The fresh or frozen PBMCs were resuspended in stem cell growth medium (CellGenix®, Freiburg, Germany) supplemented with 2.5% autoserum containing IL-2 (5 ng/mL), IL-15 (10 ng/mL), OKT3 (10 ng/mL), tacrolimus (0.02 ng/mL, Fujisawa, Japan), and dalteparin sodium (10 U/mL, Pfizer Japan). The PBMCs were cultured at a density of 10^6^/mL in 24- or 6-well plates at 37°C in 5% CO_2_ for 3 weeks. Cell cultures were split into half every 3–4 days, and fresh medium containing cytokines and reagents was added as previously described by our group [11]. The proportion of NK cells was measured before, as well as at 7, 14, and 21 days after expansion, using a flow cytometer, and eNKs obtained on day 21 were immediately used for assays or cryopreserved in liquid nitrogen. The frozen eNKs were recultured in stem cell growth medium supplemented with 5% autoserum for 48 hr, in the presence of IL-2 (500 ng/mL) and IL-15 (20 ng/mL), and then used for cytotoxicity assays after washing.

### 2.4. Lactate Dehydrogenase (LDH)-Based Cytotoxicity Assay

We measured the activity of lactate dehydrogenase (LDH), which is released into the medium when T cells are damaged and considered as an enzymatic indicator of cellular death. A Cytotoxicity LDH Assay Kit-WST (#CK12, Dojindo Laboratories, Kumamoto, Japan) was used according to the manufacturer's protocol. Briefly, NK-92MI cells were pretreated without or with Elo (0.25–20 *μ*g/mL) on 96-well *U*-bottom plates for 30 min at 37°C in 5% CO_2_, following which K562 and MM cells (10^4^/well) were added at *E : T* ratios of 1 : 1 and 2.5 : 1. Alternatively, NK-92MI/CD16a or eNKs were seeded in plates, following which target cells pretreated without or with Elo for 30 min at 4°C prior to washing and recounting were cocultured with them. The *E : T* ratios were the same in all NK cell lines, but varied from 1 : 1 to 10 : 1 for eNKs. After 4 hr of incubation, an aliquot of 100 *μ*L supernatant was transferred to a 96-well flat-bottom plate to stop the reaction. Finally, the absorption of the released LDH was determined at 490 nm using a SpectraMax® i3 microplate reader (Molecular Devices, San Jose, CA, USA). NK cell-mediated cytotoxicity was calculated as % cytotoxicity, according to the manufacturer's protocol. The supernatants after coculture were also collected and partly cryopreserved for detection of protein later.

### 2.5. Enzyme-Linked Immunosorbent Assay (ELISA) to Detect Interferon (IFN)-*γ*

To evaluate the levels of the cytokine interferon (IFN)-*γ* upon coculture of NK cell lines with target cells for 4 hr, the supernatants were quantified using ELISA. A LEGEND MAX™ Human ELISA Kit (#430107, BioLegend) containing precoated human IFN-*γ* plates was used according to the manufacturer's protocol, following which the absorbance at 450 nm was measured using a microplate reader. The IFN-*γ* concentration was analyzed based on a standard curve obtained upon serial dilution of standard protein in duplicate, using SoftMax® Pro version 6.3 (Molecular Devices).

### 2.6. FCM-Based Cytotoxicity Assay and Analysis

For the target cell killing assay, K562 and MM cells were labeled with CFSE dye (2.5 *μ*M) for 10 min in the dark, at 37°C, before being washed and recounted, and then cocultured with NK cells at different *E : T* (5 × 10^4^) ratios in a 96-well plate at 37°C in 5% CO_2_. A similar setup was created for NK-92MI/CD16a cells with K562, U266, RPMI 8226, or MM.1S cells pretreated without or with Elo (10–20 *μ*g/mL) for 30 min at 4°C, at *E : T* (10^5^) ratios of 1 : 1 and 2.5 : 1. NK-92MI cells were first pretreated without or with Elo (10–20 *μ*g/mL) for 1 hr at 37°C, and then cocultured with target cells. After 4 hr, the total cells were harvested and washed, stained for 20 min with FVD dye (1 *μ*L/mL), and then subjected to FCM. The percentage of dead target cells among CFSE^+^FVD^+^/total CFSE^+^ cells was calculated after subtracting the percentage of dead negative control cells.

In the CD107a degranulation assay, the target cells were first pretreated without or with Elo for 30 min at 4°C, washed and recounted, and then incubated with NK-92MI/CD16a cells. Additionally, NK-92MI cells were pretreated without or with Elo for 1 hr at 37°C prior to the addition of target cells. The effector and target cells were incubated for 1 hr at an *E : T* (10^5^/well) ratio of 1 : 1 in 96-well plates in the presence of an anti-CD107a at 37°C in 5% CO_2_. Each type of cell was also seeded alone in the plates as a control. Next, an inhibitor monensin was added (1 : 1000) and the cells were incubated for an additional 3 hr. After a total duration of 4 hr, the cells were collected and stained with anti-CD56 or anti-CD319 separately for 30 min at 4°C, following which the proportions of CD107a^+^CD56^+^ were detected using a flow cytometer. The expression of surface markers in NK and target cells and the purity of eNKs were also analyzed after staining with appropriate fluorescent-conjugated antibodies. All samples were analyzed using a Navios flow cytometer and Kaluza Analysis Software version 2.1 (both from Beckman Coulter).

### 2.7. Quantitative Real-Time Polymerase Chain Reaction (qPCR) for Measuring mRNA Expression in CD16-Independent NK Cells

For analysis of associated genes in NK-92MI, the mRNA expression in cells stimulated with Elo was assessed using qPCR. NK-92MI cells pretreated without or with Elo (10 *μ*g/mL) were first cultured for 1 hr in 96-well plates at 37°C in 5% CO_2_, following which target cells (10^5^/well) were added (*E : T* ratio of 10 : 1). The total cells were collected after incubation for 1, 2, 4, and 24 hr, and then washed and stored as pellets until use. NK-92MI and each target cell obtained at 0 hr were also stored as controls. Total RNA was extracted using the PureLink RNA Mini Kit (Invitrogen), and cDNA was synthesized using the High Capacity cDNA Reverse Transcription Kit (Applied Biosystems, Carlsbad, CA, USA), according to the manufacturer's protocol. qPCR was performed using a QuantStudio™ 3 Real-Time PCR System (Applied Biosystems), with coamplification of the endogenous control GAPDH gene as the standard qPCR program. Gene expression levels were determined using the TaqMan® probe-based assay (Table S1) and calculated using the 2^−*ΔΔ*Ct^ algorithm method. The relative mRNA expression levels of every gene were expressed as a ratio of the expression in the group pretreated with Elo to that in the group pretreated without Elo. The levels of mRNA expression of samples obtained at each time point were also compared to the expression of NK-92MI cells at 0 hr.

### 2.8. Statistical Analysis

All data were evaluated using Student's *t*-test. The results expressed as mean percentage ± standard deviation of independent experiments, respectively. Statistically significant was set at  ^*∗*^*P* < 0.05,  ^*∗∗*^*P* < 0.01, and  ^*∗∗∗*^*P* < 0.005.

## 3. Results

### 3.1. Characterization of Cell Surface Marker Expression in Effector and Target Cells

**Figure 1:**
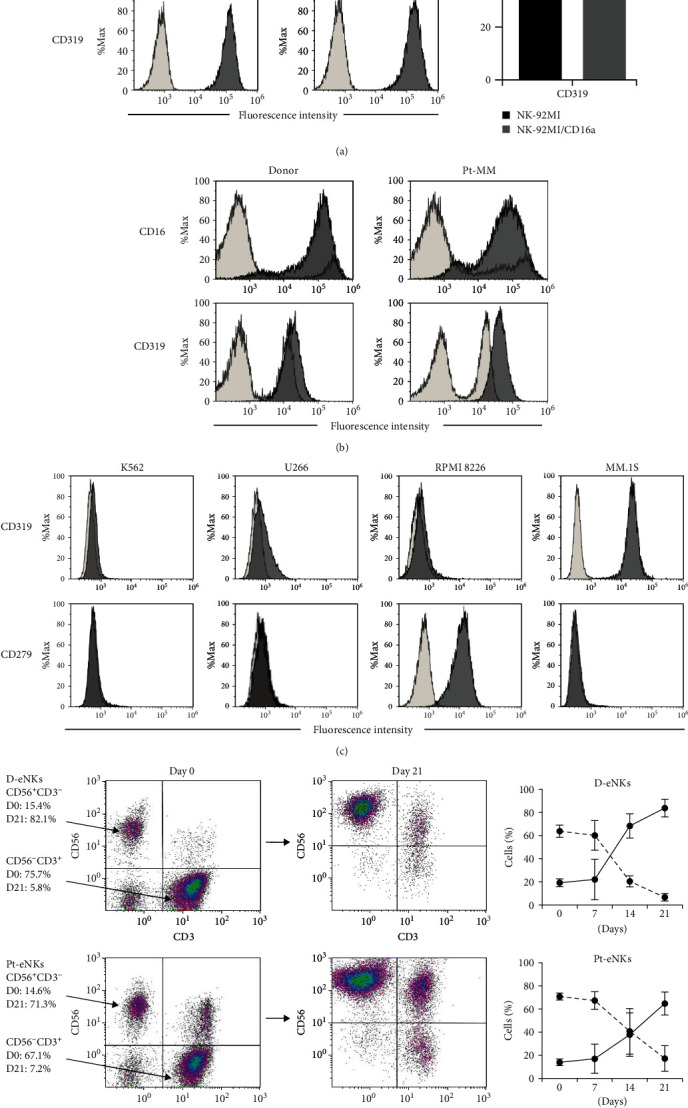
Characterization of surface marker expressions on NK effector cells and target cell lines using FCM analysis: (a) different expression of CD16 (upper panel) and CD319 (SLAMF7) (lower panel) in the two human NK cell lines, NK-92MI and NK-92MI/CD16a. No significant difference in the MFI of CD319 between the two cell lines (*n* = 5, right panel), (b) representative results of CD16 (upper panel) and CD319 (lower panel) before (light gray, day 0) and after (dark gray, day 21) expansion of fresh PBMCs from a donor (left panel) and patient (right panel), (c) CD319 (upper panel) and CD279 (PD-1) (lower panel) in target K562, U266, RPMI 8226, and MM.1S cells. Histogram overlay showing representative results for each in comparison to the control. Light gray, isotype control; dark gray, stained with anti-CD16, anti-CD319, or anti-CD279 (a)–(c), and (d) representative result of CD56^+^CD3^–^ and CD56^–^CD3^+^ before (day 0) and after (day 21; left panel) expansion of frozen PBMCs from a donor (upper panel) or patient (lower panel). Each data point represents mean ± SD of CD56^+^CD3^–^ (solid line) and CD56^–^CD3^+^ (dotted line) on eNKs from donors and patients (right panel, day 0 to day 21, *n* = 3, respectively).

CD16, CD319 (SLAMF7), or CD279 were evaluated using FCM in NK cell lines, eNKs, and target cells. Both NK-92MI and NK-92MI/CD16a cells expressed a high level of SLAMF7, with no significant difference in the mean fluorescence intensity between the two. NK-92MI/CD16a cells showed high CD16 expression, and no expression was detected in NK-92MI cells (Figure 1(a)). These two NK cell lines were found to express very high levels of CD56 and NK-activation and NK-inhibition markers, such as NKG2D, NKp46, and NKG2 by FCM analysis (data not shown). Comparing PBMCs derived from one donor and one patient sample before and after expansion, CD16 increased from 10.2% to 80.7% and from 13.2% to 67.9%, whereas SLAMF7 increased from 32.2% to 98.1% and from 38.5% to 98.7%, respectively (Figure 1(b)). MM.1S expressed high levels of SLAMF7, while U266 expressed very low levels, and K562 and RPMI 8226 showed no expression. Only RPMI 8226 were found to express PD-1, an immune checkpoint receptor, while the other cells did not (Figure 1(c)). High–purity NK cells were obtained from the PBMCs of donors and patients with MM, upon comparison with those before expansion (Figure 1(d)). NK cells from the donors and patients increased from 19.3% to 83.8% and from 14.0% to 64.7%, respectively, while CD56^–^CD3^+^ cells were clearly decreased in PBMCs from donors (63.8% ± 5.2% to 6.7% ± 3.3%) and patients (70.8% ± 3.0% to 17.3% ± 11.1%; day 0 to day 21). High levels of viable eNKs (>80%) were obtained from these samples before use in the assays. Based on these results, we concluded that NK-92MI/CD16a or eNKs can be used as effectors to evaluate NK-mediated ADCC in target cells, and the CD16-independent NK-92MI cells can be used to investigate Elo-mediated cytotoxicity in the same target cells.

### 3.2. Efficacy of NK Cell-Mediated Cytotoxicity against Target Cells Increases as the *E : T* Ratio Increases Both in NK Cell Lines and Expanded NK Cells

**Figure 2:**
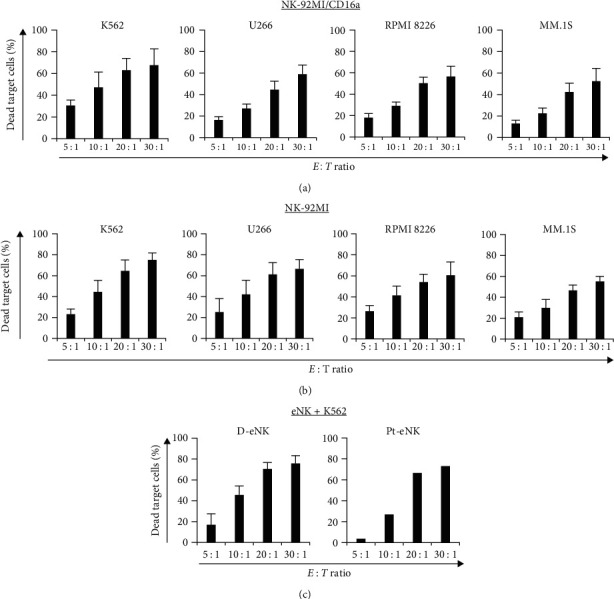
FCM-based assessment of the cytotoxicity of NK effector (E) cells against target (T) cells at different *E : T* ratios: (a) NK-92MI/CD16a (CD16-dependent) or (b) NK-92MI (CD16-independent) cells were incubated for 4 hr with the CFSE (2.5 *µ*M)-prelabeled K562, U266, RPMI 8226, and MM.1S cells. The percentage of dead target cells (CFSE^+^FVD^+^) was analyzed among the total FVD dye-labeled cells (*n* = 5), and (c) freshly eNKs were collected and washed, and then were cocultured with CFSE-labeled K562. The percentage of eNK (day 21) was 75.4% ± 11.3% (D-eNK, left panel, *n* = 3) and 69.9% (Pt-eNK, right panel, *n* = 1). Results are presented as mean ± SD of independent experiments, respectively. The percentage of labeled control cells was subtracted in all assays.

To investigate the NK cell-mediated cytotoxicity, the population of dead target cells, indicated by CFSE^+^FVD^+^, was analyzed using FCM.  Figure 2(a) shows the results obtained upon coculture of NK-92MI/CD16a cells with each target cell for 4 hr, at multiple *E : T* ratios. The cytotoxicity increased from 30.5% to 67.7% (K562), 16.4% to 58.9% (U266), 18.0% to 56.6% (RPMI 8226), and 13.0% to 52.4% (MM.1S), when the *E : T* ratio increased from 5 : 1 to 30 : 1. The NK-92MI cells showed similar levels of cytotoxicity against these target cells as those shown by NK-92MI/CD16a, that is, 75.0% ± 6.7% in K562 and 55.2% ± 4.7% in MM.1S cells, at an *E : T* ratio of 30 : 1 (Figure 2(b)). Both the NK cells showed very high cytotoxicity at a high *E : T* ratio against K562, which is the only HLA-negative cell line among the target cell lines (in our analysis by FCM, data not shown); these results possibly suggest that HLA class I-positive cells are less susceptible to NK cell-mediated death than HLA-negative cells [23].  Figure 2(c) shows the cytotoxicity of fresh eNK effector cells against K562. The cytotoxicity of eNKs from the donors increased significantly from 16.7% ± 10.5% to 75.7% ± 7.5% when the *E : T* ratio increased from 5 : 1 to 30 : 1, although it was not as high as that of NK-92MI/CD16a. Moreover, eNKs derived from a patient with MM showed that the cytotoxicity reached up to 73% at an *E : T* ratio of 30 : 1. From these results, we hypothesized that the NK-mediated ADCC in the presence of Elo may be more effective and sensitive at a lower *E : T* ratio.

### 3.3. Elo Increases CD16-Dependent NK-92MI/CD16a- and Expanded NK Effector Cell-Mediated ADCC Activity Only against SLAMF7-Positive Target Cells

**Figure 3:**
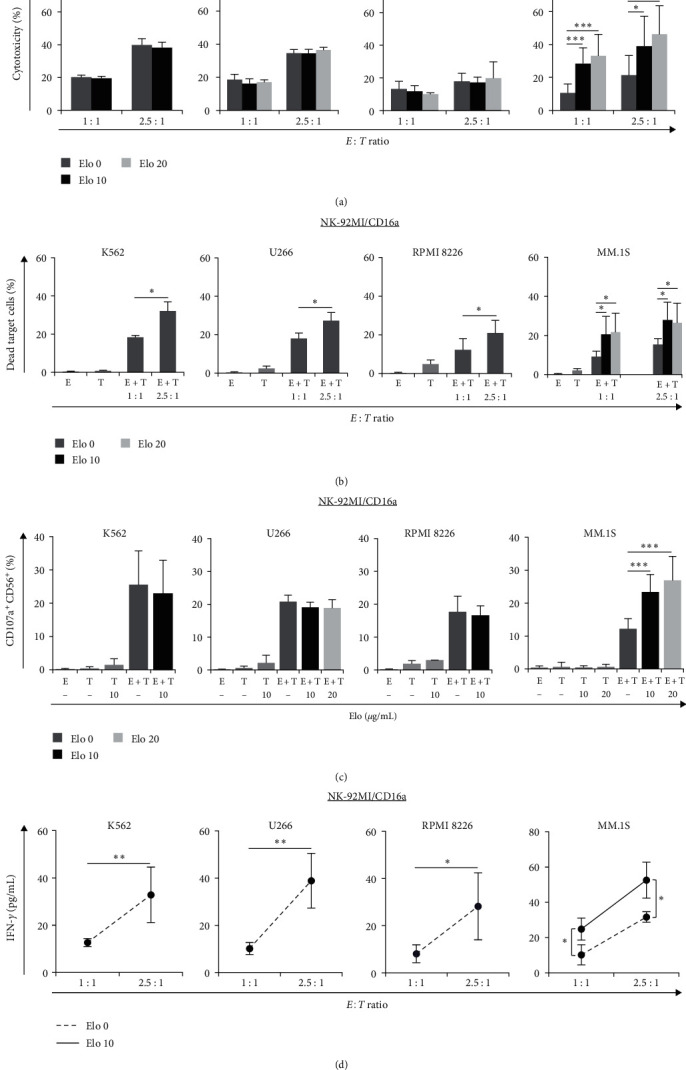
NK-92MI/CD16a (E, CD16-dependent) cell-mediated cytotoxicity against K562, U266, RPMI 8226, and MM.1S target (T) cells pretreated without or with Elo (10 *μ*g/mL and/or 20 *μ*g/mL), and their IFN-*γ* production: (a) LDH assay showing the cytotoxicity of NK-92MI/CD16a against various target cells, (b) FCM analyses of dead target cells (CFSE^+^FVD^+^) at *E : T* ratios of 1 : 1 and 2.5 : 1. MM.1S cells were also pretreated with Elo and incubated with NK-92MI/CD16a after washing, (c) degranulation of NK-92MI/CD16a was assessed as CD107a^+^CD56^+^ expression on various target cells, when target cells were pretreated without (−) or with Elo (*E : T* = 1 : 1), using FCM assay. gray bars, and (d) ELISA-based detection of IFN-*γ* production after coculture with NK-92MI/CD16a and target cells, pretreated without (dotted line) or with (solid line) 10 *µ*g/mL of Elo. Results for (a)–(d) are presented as mean ± SD of three to five independent experiments.  ^*∗*^*P* < 0.05,  ^*∗∗*^*P* < 0.01, and  ^*∗∗∗*^*P* < 0.005.

Subsequently, we investigated the effect of NK-mediated ADCC activity on CD16-positive cell lines or eNKs pretreated without or with Elo using the LDH assay and FCM analysis. The LDH assay results indicated that NK-92MI/CD16a-mediated cytotoxicity against K562, U266, and RPMI 8226 did not change regardless of the presence or absence of Elo, while maintaining the *E : T* ratio-dependent effect (Figure 3(a)). In case of MM.1S, however, NK cell-mediated cytotoxicity increased significantly upon pretreatment with Elo compared with that pretreated without Elo. Additionally, NK-92MI/CD16a-mediated cytotoxicity increased significantly depending on the concentration of Elo added to MM.1S (Figure S1(a)). The NK-92MI/CD16a-mediated cell death was also observed to be higher at an *E : T* ratio of 2.5 : 1 than that at a ratio of 1 : 1 in K562, U266, and RPMI 8266 using FCM analysis (Figure 3(b)). The cytotoxicity mediated by NK-92MI/CD16a cells was significantly higher in MM.1S pretreated with Elo than in cells pretreated without Elo. The LDH assay was more sensitive than FCM in identifying subtle changes. In addition, NK-92MI/CD16a cells presented significantly increased expression of CD107a^+^CD56^+^ against MM.1S cells upon pretreatment with Elo compared to cells pretreated without Elo (Figure 3(c), *P* < 0.005 both with 10 and 20 *μ*g/mL Elo). In contrast, in SLAMF7-negative K562, U266, and RPMI 8226, there was no significant difference between cells pretreated without and with Elo, with no increase in Elo-mediated ADCC.

IFN-*γ* is a major proinflammatory factor and regulatory cytokine produced by activating effector immune cells such as T and NK cells [24]. As shown in Figure 3(d), IFN-*γ* production from the collected supernatant was significantly increased at an *E : T* ratio of 2.5 : 1 compared to that at a ratio of 1 : 1 for K562, U266, and RPMI 8226. In case of SLAMF-positive MM.1S cells, however, pretreatment with Elo significantly enhanced the IFN-*γ* production at *E : T* ratios of 1 : 1 and 2.5 : 1 (24.8 ± 6.3 and 52.6 ± 10.2 pg/mL, respectively), compared to pretreatment without Elo at the *E : T* same ratios (10.3 ± 5.7 and 31.7 ± 3.0 pg/mL, respectively; 1 : 1, *P*=0.024 and 2.5 : 1, *P*=0.018). We found that incubation with NK-92MI/CD16a cells simultaneously or pretreated with Elo followed by the addition of target cells did not increase NK cell-mediated cytotoxicity, suggesting that activation of receptor CD16a by stimulation of mAb regents appears to impair their function and downregulate the effect of ADCC [25, 26].

**Figure 4:**
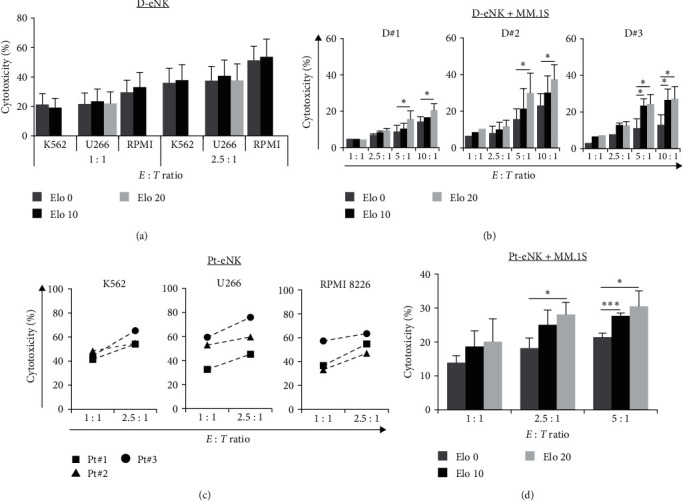
LDH assay for the assessment of the eNK (E, frozen) cell-mediated cytotoxicity pretreated without or with Elo (10 *μ*g/mL and/or 20 *μ*g/mL) against K562, U266, RPMI 8226 (RPMI), and MM.1S target (T) cells: (a) results show the cytotoxicity when D-eNKs were cocultured with K562, U266, and RPMI pretreated without or with Elo (*n* = 3), (b) D-eNK-mediated cytotoxicity against MM.1S pretreated without or with Elo (*E : T* ratios of 1 : 1, *n* = 1; 2.5 : 1 to 10 : 1, *n* = 3). The percentages of eNKs were 55.4% ± 10.0%, 61.2% ± 7.2%, and 63.7% ± 5.9% for donor no. 1 (D#1), no. 2 (D#2), and no. 3 (D#3), respectively, (c) cytotoxicity of Pt-eNKs against K562, U266, and RPMI 8226. The percentages of eNKs were 44.6%, 68.7%, and 79.0% for patient no. 1 (Pt#1, ▪), no. 2 (Pt#2, ▴), and no. 3 (Pt#3, •), respectively, and (d) Pt-eNK cell-mediated cytotoxicity against MM.1S cells pretreated without or with Elo at different *E : T* ratios (*n* = 3). Results are presented as mean ± SD of independent experiments, respectively.  ^*∗*^*P* < 0.05 and  ^*∗∗∗*^*P* < 0.005.

Next, we investigated the cytotoxicity of eNKs against target cells using an LDH assay. Frozen eNKs were cocultured with target cells after recovering NK activity in the presence of IL-2 and IL-15 prior to the assay. Donor-eNK (D-eNK)-mediated cytotoxicity against K562, U266, and RPMI 8226 increased depending on the *E : T* ratio, and there was no significant difference between cells treated without and with Elo in each group (Figure 4(a)). We also incubated D-eNKs with MM.1S cells at increasing *E : T* ratios of 1 : 1 to 10 : 1 (Figure 4(b)), which indicated that the higher *E : T* ratios resulted in increased eNK cell-mediated cytotoxic effect on MM.1S; moreover, pretreatment with Elo significantly increased eNK-mediated cytotoxicity at ratios of 5 : 1 and 10 : 1 compared with that observed in the cells pretreated without Elo. Overall, D-eNK-mediated cytotoxicity against MM.1S showed a concentration-dependent increase upon addition of Elo (Figure S1(b)). The cytotoxic effect of patient-eNKs (Pt-eNK) at an *E : T* ratio of 2.5 : 1 was higher than that at a ratio of 1 : 1, although the extent was slightly different in K562, U266, and RPMI 8226 (Figure 4(c)). Upon studying the effects of Pt-eNKs on MM.1S pretreated with Elo at increasing *E : T* ratios from 1 : 1 to 5 : 1 (Figure 4(d)), the Pt-eNK-mediated cytotoxicity was similar between cells that received pretreatment with Elo at 10 (*P*=0.002) and 20 *μ*g/mL (*P*=0.029), with both being higher than that observed with cells pretreated without Elo.

These data indicate that the significant antitumor effects of eNKs; however, the addition of Elo enhances eNK-mediated ADCC only against SLAMF7-positive MM cells, similar to that seen in the case of NK-92MI/CD16a. A higher *E : T* ratio and/or higher Elo concentration than that of the NK cell line may be more effective in a case where the percentage of eNKs is lower.

### 3.4. Elo Enhances CD16-Independent Cytotoxicity of NK-92MI Cells against Both SLAMF7-Negative- and -Positive Target Cells

**Figure 5:**
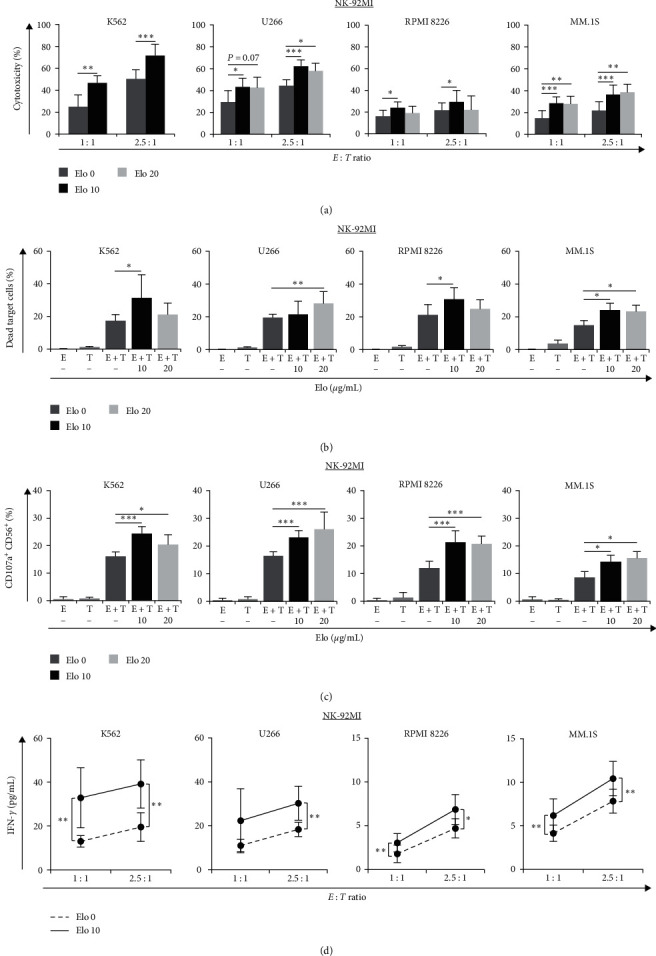
NK-92MI (E, CD16-independent) cell-mediated cytotoxicity pretreated without or with Elo (10 *μ*g/mL and/or 20 *μ*g/mL) against K562, U266, RPMI 8226, and MM.1S target (T) cells, and their IFN-*γ* production: (a) LDH assay showing the cytotoxicity of NK-92MI pretreated without or with Elo (37°C, 30 min) against various target cells (*E : T* ratios of 1 : 1 and 2.5 : 1), (b) FCM analyses of dead target cells (CFSE^+^FVD^+^) at an *E : T* ratio of 2.5 : 1, when NK-92MI were pretreated without (−) or with Elo (37°C, 1 hr). All target cells were prelabeled with CFSE (2.5 *μ*M) before being washed and recounted, and then incubated with the effector cells, (c) degranulation of NK-92MI was assessed as CD107a^+^CD56^+^ expression on various target cells, when NK-92MI were pretreated without (−) or with Elo (*E : T* = 1 : 1), using FCM assay, and (d) ELISA-based detection of IFN-*γ* production after coculture of NK-92MI with target cells without (dotted line) or with (solid line) 10 *μ*g/mL of Elo. Results in (a)–(d) are presented as mean ± SD of four to five independent experiments for every assay.  ^*∗*^*P* < 0.05,  ^*∗∗*^*P* < 0.01, and  ^*∗∗∗*^*P* < 0.005.

Given the observed CD16-dependent NK cell-mediated cytotoxicity against target cells, we examined whether the addition of Elo also increased the activity of NK-92MI cells, which are without CD16, against K562 and MM cells. NK-92MI is an IL-2-independent cell line derived from NK-92 (#CRL-2407, ATCC), which has been transfected with human IL-2 cDNA and stably cultured. NK-92MI expressed surface markers similar to the parental NK-92 and showed high SLAMF7 expression in our experiments using FCM analysis. NK-92MI cell-mediated cytotoxicity against SLAMF7-negative target cells was not significantly different irrespective of whether Elo was added simultaneously with the effector to the target cells, or target cells were pretreated first with Elo followed by incubation with effector cells, compared to that observed in the absence of Elo. However, in the LDH assay, pretreatment of NK-92MI with Elo, followed by incubation with each target cell yielded significant results (Figure 5(a)). NK-92MI alone showed increasing cytotoxicity against the target cells as the *E : T* ratio increased. The addition of Elo directly activated NK cells and resulted in significantly increased cytotoxicity, not only against SLAMF7-positive MM.1S cells but also against SLAMF7-negative K562, U266, and RPMI 8226 cells, compared to cells pretreated without Elo. The effect of Elo pretreatment on the cytotoxicity of NK-92MI against MM.1S was also Elo concentration-dependent (Figure S1(c)). The cytotoxic effect of NK-92MI against MM.1S cells seemed to be influenced by the *E : T* ratio and Elo concentration. There was no significant difference in the cytotoxicity of the effector cells, in the presence or absence of the negative control IgG1 mAb (Figure S1(d)). Moreover, Elo did not enhance the cytotoxicity of SLAMF7-positive NK cells against autologous NK-92MI cells (Figures S1(d) and S2).  Figure 5(b) shows similar results for NK-92MI against target cells at an *E : T* ratio of 2.5 : 1, in which effector cells pretreated first with Elo were more effective than those pretreated without Elo in the killing of target cells. NK-92MI cells displayed weaker but significant CD107a expression (Figure 5(c)) compared to that of NK-92MI/CD16a cells. After pretreatment of NK-92MI cells with Elo and subsequent culture with the target cells, CD107a^+^CD56^+^ expression was significantly increased compared to cells pretreated without Elo. Similar effects were observed with pretreatment with Elo at 10 and 20 *μ*g/mL in this degranulation assay.

As an indicator of NK cell activity, IFN-*γ* was also measured in NK-92MI cells. As shown in Figure 5(d), IFN-*γ* was clearly produced by increasing the *E : T* ratio, and pretreatment with Elo also significantly enhanced IFN-*γ* levels in each target cell, compared to cells pretreated without Elo. IFN-*γ* levels and cytotoxic effects were lower in RPMI 8226 and MM.1S in all analyses and assays, which could probably be owing to these two cell lines having their own special markers such as PD-1 or SLAMF7; however, they increased significantly after addition of Elo.

**Figure 6:**
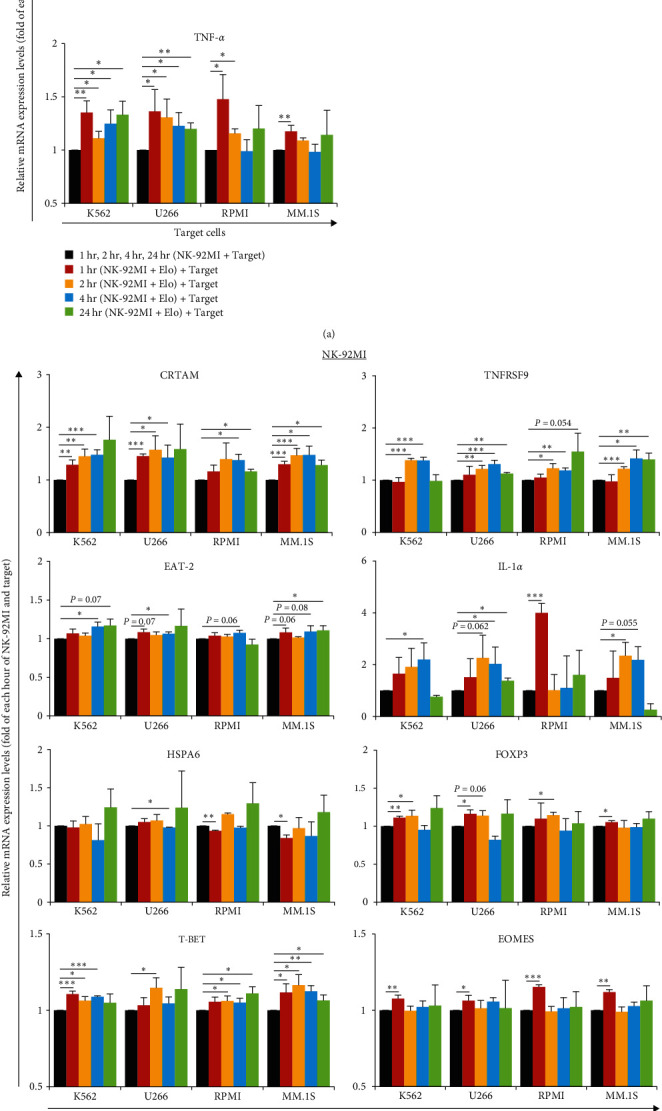
qPCR analysis of the relative mRNA expression of GZMB, PRF1, TNF-*α* (a), CRTAM, TNFRSF9, EAT-2, IL-1*α*, HSPA6, FOXP3, T-BET, and EOMES and (b) in NK-92MI (E, CD16-independent) cells pretreated without or with Elo (10 *μ*g/mL, 37°C, 1 hr), and then cocultured with K562, U266, RPMI 8226 (RPMI), and MM.1S target (T) cells (*E : T* ratio of 10 : 1) for 1, 2, 4, and 24 hr. Gene expression levels are expressed as the ratio of the expression in the group pretreated with Elo to that in the group pretreated without Elo (black bar). The Elo-pretreated groups are shown at the time points of 1 hr (red), 2 hr (orange), 4 hr (blue), and 24 hr (green). Results are presented as mean ± SD of four independent experiments for every target cell.  ^*∗*^*P* < 0.05,  ^*∗∗*^*P* < 0.01, and  ^*∗∗∗*^*P* < 0.005.

Granzyme B (GZMB), perforin 1 (PRF1), and tumor necrosis factor (TNF)-*α* are representative functional molecules of NK cell-mediated cytotoxicity [27, 28]. We further assessed whether the presence of Elo could enhance the antitumor effect of NK-92MI by measuring the expression of these genes. We first pretreated NK-92MI without or with Elo, cocultured them with target cells, and finally measured the mRNA expressions of these gene after 1, 2, 4, and 24 hr (*E : T* ratio of 10 : 1). As shown in Figure 6, the expression of various genes in the E and T group in the absence of Elo was measured at each hour and used as control values. It was observed that GZMB expression increased significantly from 4 to 24 hr and PRF1 expression increased slowly at 24 hr in all target cells; TNF-*α* expression increased in K562 and U266 over the 1–24 hr time period, while RPMI 8226 and MM.1S reached the maximum expression at 1 hr of coculture with Elo-treated NK-92MI (Figure 6(a)). Upon taking the expression of NK-92MI cells at 0 hr as the reference, similar results were observed, and are shown in Figure S2(a). These results demonstrated that in the presence of Elo, the increased mRNA expression of GZMB and TNF-*α* happened earlier compared with that observed in the absence of Elo. On the contrary, the increase of PRF1 appeared to occur later; however, this could be attributed to the possibility of transcription into the protein after 24 hr of coculture. GZMB and TNF-*α* seemed to be highly involved in the NK-92MI cell-mediated cytotoxicity against target cells after Elo stimulation. As observed in the previous experiments, the mRNA expression levels of the genes also indicated that effects of NK-92MI were more pronounced against K562 and U266, than against RPMI 8226 and MM.1S, possibly because K562 and U266 do not have unique markers.

### 3.5. Elo Enhances CD16-Independent NK-92MI Cell-Mediated Cytotoxicity against Target Cells via Activation of Several NK Cell-Enhancing Genes, Including Cytotoxic and Regulatory T Cell Molecule (CRTAM) and TNF Receptor Superfamily Member 9 (TNFRSF9)

Besides GZMB, PRF1, and TNF-*α*, we assessed the expression of several other genes possibly involved in NK-92MI-mediated cytotoxicity against target cells in the presence of Elo using qPCR assays to confirm their contribution (Figure 6(b)). CRTAM (also known as CD355), a cell surface receptor, is a member of the immunoglobulin superfamily that plays an important role in the antitumor activity of NK cells and CD8^+^ T lymphocytes [29]. TNFRSF9 (also known as 4-1BB or CD137) belongs to the TNF receptor family, and its potential role is to increase the function of the effectors by regulating costimulatory molecules with T cells [30], which has been introduced into the chimeric antigen receptor structure transduced T-cell to further enhance cytotoxic T-cell function in cancer immunotherapy [31]. In all target cells, the mRNA expression of CRTAM and TNFRSF9 increased significantly in NK-92MI pretreated with Elo for 1–4 hr, compared with that observed in the cells not pretreated with Elo. In addition, the expression was continuously and significantly elevated in SLAMF7-positive MM.1S, even at 24 hr. The SH2 domain-containing 1B (EAT-2) is an intracellular adapter related to SLAM-associated protein that regulates signal transduction through receptors to control the effects of NK cells [19, 32]. EAT-2 expression increased significantly in K562 and U266, and an increasing trend was observed in RPMI 8226 (*P*=0.06) and MM.1S (*P*=0.08) at 4 hr in the Elo-treated group. IL-1*α* is a pleiotropic cytokine involved in various immune responses, inflammatory processes, and hematopoiesis [33]. In the presence of Elo, the expression of IL-1*α* was continuously elevated up to 4 hr, and showed a highly drastic increase at 24 hr. In addition, the level increased significantly in K562, U266, and MM.1S (*P*=0.055), while it reached a maximum level at 1 hr in RPMI 8226, compared to that in the control group. Similar results for the expression of these four genes are shown in Figures S2(a) and S2(b), in which only RPMI 8226 were found to express TNFRSF9.

Next, the expression of four other protein-coding genes was verified using qPCR. Heat shock protein family A member 6 (HSPA6, also known as HSP70B), an immune-related gene, has been reported to be essential for improving cell survival under various stress factors [34]. HSPA6 expression in the Elo-pretreated group decreased significantly in U266 at 4 hr, and in RPMI 8226 and MM.1S after 1 hr of coculture with NK-92MI; however, no such significant difference was observed in K562. Forkhead box P3 (FOXP3) is a transcriptional regulator that plays a crucial role in maintaining the stability and regulating the suppressive functions of Tregs in the immune system [35]. We observed that in the group pretreated with Elo, FOXP3 expression did not decrease much and increased significantly at 1 hr for K562, U266, and MM.1S, and at 2 hr for RPMI 8226; however, in U266, the expression decreased significantly at 4 hr (*P*=0.023). T-box transcription factor 21 (T-BET) and eomesodermin (EOMES) are members of the T-box gene family and are crucial transcription factors involved in the regulation of NK cell-mediated immune responses to infections and cancer [36]. A significant increase was observed in T-BET expression in the presence of Elo, compared with that in the control group without Elo, during the 1–4 hr time-period in K562, RPMI 8226, and MM.1S; this increase persisted until 24 hr for RPMI 8226 and MM.1S. In U266 cells, the increase was observed at 2 hr. In contrast, EOMES expression in the Elo group increased significantly only at 1 hr compared with that in the control group (Figure 6(b) and Figures S2(b) and S2(c)).

We also analyzed the expression of UL16-binding protein 1 (ULBP1). It is a ligand for NKG2D, an immune system-activating receptor of NK and T cells. Binding of this ligand to the NKG2D receptor results in the activation of several signaling pathways and stimulates NK cell-mediated cytotoxicity [37]. We found that although ULBP1 expression was positive in all target cells, it was barely detectable after 1 hr of coculture with effect cells, owing to which the effect of Elo pretreatment on the expression levels could not be examined. We hypothesized that ULBP1 on the surface of target cells was quickly recognized by NKG2D on NK-92MI cells, which led to enhanced NK cell-mediated cytotoxicity followed by a decreased in ULBP1 expression in the absence of Elo.

## 4. Discussion

Recent advances in treatment methods have made it possible for patients with MM to maintain their quality of life and to prolong survival. In particular, mAb-based immunotherapies such as Elo have been used as salvage therapies for patients with RRMM. Moreover, the immunomodulatory and antitumor properties of NK cells have also attracted attention in cancer immunotherapy [10]. In this context, owing to its high levels of expression on plasma and NK cells, the cell surface transmembrane glycoprotein SLAMF7 serves as an interesting target [14, 15].

Regarding the mechanism by which Elo targets SLAMF7 and induces ADCC, NK cell-mediated cytotoxicity is caused by crosslinking of the NK cell receptor CD16a to the Fc region of Elo, which binds to SLAMF7 on the MM cell membrane [14]. Our findings are consistent with those of previous studies demonstrating the effect of Elo on NK cells *via* CD16-mediated ADCC [17, 19, 20]. In this study, NK-92MI/CD16a and activated eNK cells, including those recovered from freezing, showed significant antitumor effects in an *E : T* ratio-dependent manner. The combination of Elo with NK cells proved effective in killing SLAMF7-positive myeloma cells in an Elo concentration-dependent manner, while Elo also strongly promoted NK-92MI/CD16a-mediated cell degranulation and IFN-*γ* production. Our observations showed no change in K562, U266, and RPMI 8226 cells without and with Elo pretreatment since SLAMF7 is not expressed in these cells (Figure S3(a)). CD16-dependent NK cells alone were able to exert sufficient antitumor effects with an increase in the *E : T* ratio. This indirect effect depends on the efficient interaction between Fc-CD16a and NK cells [13]. Combination therapy with mAbs and autologous eNKs is safe and has been shown to enhance the therapeutic activity of NK cells in patients with relapsed CD20-positive lymphoma *in vivo* [11, 12]. Similarly, a clinical trial of autologous eNKs in combination with Elo-containing chemotherapy is currently underway for patients with RRMM [18]. Elo also has the potential to eliminate SLAMF7^+^CD8^+^ regulatory T cells, and is now being considered in therapies that target the suppressive tumor microenvironment by combining immunomodulatory agents. Combination of mAbs enhances the effect of ADCC by genetically engineering NK cells expressing a high-affinity and noncleavable CD16 variant. Moreover, cytokine expression in combination with priming agents in next-generation NK cell products can be expected to improve NK cell surface CD16 expression and persistence of NK function. Although further studies are needed to optimize and maintain the NK cell activation status for maximum antitumor efficacy *in vivo*, combination therapy incorporating Elo with NK cells and other agents that can potentiate NK cell functions is a promising strategy for MM treatment [38, 39].

Another possible mechanism for the antitumor effect of Elo is its binding to SLAMF7 on NK cell membranes, leading to direct activation of NK cells without CD16 dependance [19, 20]; this mechanism was the primary focus of the current study. Our results showed that NK-92MI activity could be directly promoted by pretreatment with Elo, which resulted in an enhanced cytotoxic effect on SLAMF7-positive MM.1S as well as SLAMF7-negative K562, U266, and RPMI 8226 cells compared with that achieved with untreated cells. In NK-92MI cells, pretreatment with Elo resulted in significantly higher IFN-*γ* secretion and CD107a^+^56^+^ detection than that observed without any pretreatment. Binding of Elo to SLAMF7 on NK-92MI cells directly increased GZMB and TNF-*α* expression, which is consistent with the findings of previous studies [19, 28]; however, it did not cause any change in the expression of PRF1. These findings are supported by the observations that Elo-pretreated NK cells eliminate tumor cells through the release of granzymes and perforin, thereby producing IFN-*γ* and TNF-*α* as important cytokines that modulate the immune response and induce apoptosis in target cells. The results of IL-1*α* expression may indicate that IL-1*α* is on the pathway that activates and releases TNF-*α* in response to cell injury, thus inducing apoptosis [33, 40]. Notably, similar to the results obtained for TNF-*α*, increases in IL-1*α* expression were also observed and were significantly different in RPMI 8226 (from as early as 1 hr onwards), which may be associated with PD-1 expression in this cell line. Therefore, it is conceivable that the timing of therapeutic effects may differ for MM cells with specific genes.

Collins et al. [19] reported that pretreatment of human NK cells with Elo for 72 hr enhanced the cytotoxicity of SLAMF7-negative K562 cells. Consistent with previous reports, pretreatment with Elo enhanced NK cell-mediated cytotoxicity in SLAMF7-negative target cells *via* Elo-SLAMF7 interactions independent of ADCC. In contrast, Pazina et al. [20] showed that Elo has no effect on SLAMF7-negative target cells in CD16-independent SLAMF7^+^ NK cells. This difference could be attributed to the assay method, including incubation time (10 or 24 hr), and whether pretreatment with Elo was performed or not. We used NK cell lines with or without CD16a and Elo expressed with Fc region, which is different from the Fc-mutant form of Elo used in other studies [20]. Our results indicate that SLAMF7 is essential for NK cells and that 4 hr of coculture is effective against both SLAMF7-negative and SLAMF7-positive target cells; however, prestimulation with Elo is an important prerequisite. In the ADCC assay of NK-92MI/CD16a, sufficient cytotoxic effects were achieved even at low *E : T* ratios and Elo concentrations. Notably, the effects of Elo on NK-92MI should also be considered in terms of the adequacy of Elo concentration, *E : T* ratio, and prestimulation time. In this study, we observed that although limited by the *E : T* ratio, the LDH assay can be easily performed with fewer cells and is more sensitive than FCM assay for assessing NK cell-mediated cytotoxicity.

Several clinical trials of combination therapy for patients with MM have shown that Elo is ineffective as a single agent; however, it shows antimyeloma effects in combination with lenalidomide/dexamethasone or bortezomib, resulting in improved patient survival, which is further enhanced by pretreatment with lenalidomide or bortezomib [41, 42]. Lenalidomide administration has been known to cause the degradation of transcription factors in MM, while increasing the levels of IL-2 in T cells [43]. IL-2 is an important cytokine essential for NK cell proliferation, survival, and cytotoxicity. In addition, coculture of Elo with human PBMCs in the presence of IL-2 for 24–72 hr leads to NK cell activation, which induces an effective killing response in MM cells [20, 28]. Therefore, we speculate that Elo-mediated promotion of NK cell activity may occur by enhancing the sensitivity of the IL-2 receptor in NK-92MI cells. Combination of Elo with NK cells expanded with the addition of IL-2 and IL-15 may improve cellular immunotherapy against MM [12, 38].

In this study, the expression levels of CRTAM, TNFRSF9, EAT-2, FOXP3, T-BET, and EOMES were upregulated within approximately 4 hr in the cells pretreated with Elo. CRTAM is expressed only on activated NK and T cells, and the interaction of CRTAM with its ligand has been shown to promote NK cell-mediated cytotoxicity and IFN-*γ* levels [29, 44]. TNFRSF9 (CD137) also functions as a positive immune checkpoint, and there is potential for therapeutic synergy upon combining CD137 agonists and tumor-targeting antibodies [45]. In this study, TNFRSF9 expression increased in all target cells at 2 hr after Elo pretreatment, suggesting that TNFRSF9 induces the tumoricidal activity of the effector when Elo interacts with NK cells. However, whether TNFRSF9 expression was associated with PD-1 in RPMI 8226 remained unclear (Figure S2(b)). GZMB, IFNG, CRTAM, and TNFRSF9 are upregulated in NK cells after vaccination or stimulation with K562 [46]. Consistent with previous reports, our results showed no detectable expression of EAT-2 in K562 and MM cell lines. Enhanced NK cell-mediated cytotoxicity is associated with increased activation within NK cells, signaling through interactions with Elo, and the binding of the SLAM-related adapter protein EAT-2. NK cell-mediated cytotoxicity against these target cells appears to depend on the differential expression of the EAT-2 intermediate in the required signaling pathway [20, 47].

HSPA6 is involved in the biological functions of cell proliferation, migration, and invasion of tumor cells [48], although its exact function remains unclear in this study. CD4^+^FOXP3^+^ Tregs are highly abundant in the bone marrow of patients with MM and are involved in the dissemination and disease progression [49]. On the contrary, FOXP3 has also been reported to suppress the expression of IL-2 and IFN-*γ* by interacting with other transcriptional factors [49, 50]. FOXP3 expression with inhibitory function increased at 1 hr. It is possible that the addition of Elo restores antitumor immunity by inhibiting or disrupting the interactions of FOXP3 with other transcription factors [50], thereby increasing IFN-*γ* production of NK cells. However, it is not known whether FOXP3 is directly linked to the inhibition of proinflammatory genes. Our findings may explain why T-BET and EOMES genes play regulatory and complementary roles, since EOMES induces early NK cell function, T-BET modulates NK cell-mediated cytotoxicity [51, 52], and Elo stimulation further increases the antitumor effects of NK cells. In addition, overexpression of T-BET or EOMES has been shown to increase NK functionality and IFN-*γ* production in NK cells [51]. Notably, improved survival associated with higher T-BET levels in NK cells resulted in a reduction in non-relapse mortality with respect to outcomes after hematopoietic stem cell transplantation, suggesting that sustained expression of T-BET and EOMES in NK cells may be involved in defending against tumors [53], and that Elo-mediated therapy may contribute to preventing the development of posthematopoietic stem cell transplantation complications in MM. The NK-enhanced genes identified in this study by Elo stimulation may be recognized as NK cell activation factors. This study found that Elo directly activates NK cells; however, this is limited only to SLAMF7-positive NK cell lines *in vitro*; further verifications using primary cells are needed in future studies. To better explain which of the many factors upregulated by Elo are mainly responsible for the beneficial effects on NK cell-medicated cytotoxicity, further mechanistic studies of these factors and their relationship with the antitumor effects of Elo-mediated CD16-independent NK cells are required. These may lead to the wide use of NK cell-based combination antitumor immunotherapy for hematological malignancies, including patients with MM [54].

## 5. Conclusions

In conclusion, we demonstrated that as an immune-stimulatory antibody, Elo directly enhances the antitumor effects of CD16-independent NK cells. Our findings suggest that the genes CRTAM, TNFRFS9, EAT-2, IL-1*α*, FOXP3, T-BET, and EOMES are associated with the upregulation of NK-92MI cell-mediated cytotoxicity against both SLAMF7-positive and SLAMF7-negative myeloma cells upon Elo prestimulation (Figure S3(b)).

## Data Availability

Data used to support the findings of this study are available from the corresponding author upon request.

## Abbreviations

Elo: Elotuzumab
ADCC: Antibody-dependent cellular cytotoxicity
SLAMF7: Signaling lymphocytic activation molecule family 7
HLA: Human leukocyte antigen
CD: Cluster of differentiation
PBMCs: Peripheral blood mononuclear cells
D-eNKs: Donor-expanded natural killer cells
Pt-eNKs: Patient-expanded natural killer cells
*E : T* ratio: Effector to target cell ratio
FCM: Flow cytometry
MFI: Mean fluorescence intensity
CFSE: Carboxyfluorescein diacetate succinimidyl ester
IFN-*γ*: Interferon gamma
GZMB: Granzyme B
PRF1: Perforin 1
TNF-*α*: Tumor necrosis factor-alpha
CRTAM: Cytotoxic and regulatory T cell molecule
TNFRSF9: TNF receptor superfamily member 9
EAT-2: SH2 domain-containing 1B
IL-1*α*: Interleukin-1 alpha
HSPA6: Heat shock protein family A member 6
FOXP3: Forkhead box P3
T-BET: T-box transcription factor 21
EOMES: Eomesodermin.

## Supplementary Materials

Supplementary Materials Table S1: human TaqMan® primer–probe assay ID for qPCR. Figure S1: LDH assay analysis showing the NK cell-mediated cytotoxicity against target cells pretreated without or with control/different concentrations of Elo antibody. Figure S2: qPCR assay-based assessment of relative mRNA expression of genes after coculture of target cells with NK-92MI cells pretreated without or with Elo, compared to that the expression in NK-92MI cells at 0 hr. Figure S3: schematic images showing the effects of Elo on NK cell-mediated ADCC and direct promotion of NK cell (CD16-independent) activity both against SLAMF7^+/–^ and HLA class I^+/–^ target cells.
