# Supplementary figures and images for: Elotuzumab Enhances CD16-Independent NK Cell-Mediated Cytotoxicity against Myeloma Cells by Upregulating Several NK Cell-Enhancing Genes
Source: J Immunol Res. 2024 Feb 27;2024:1429879. doi: 10.1155/2024/1429879 (PMC10914431; doi:10.1155/2024/1429879)

**Supplementary Materials**


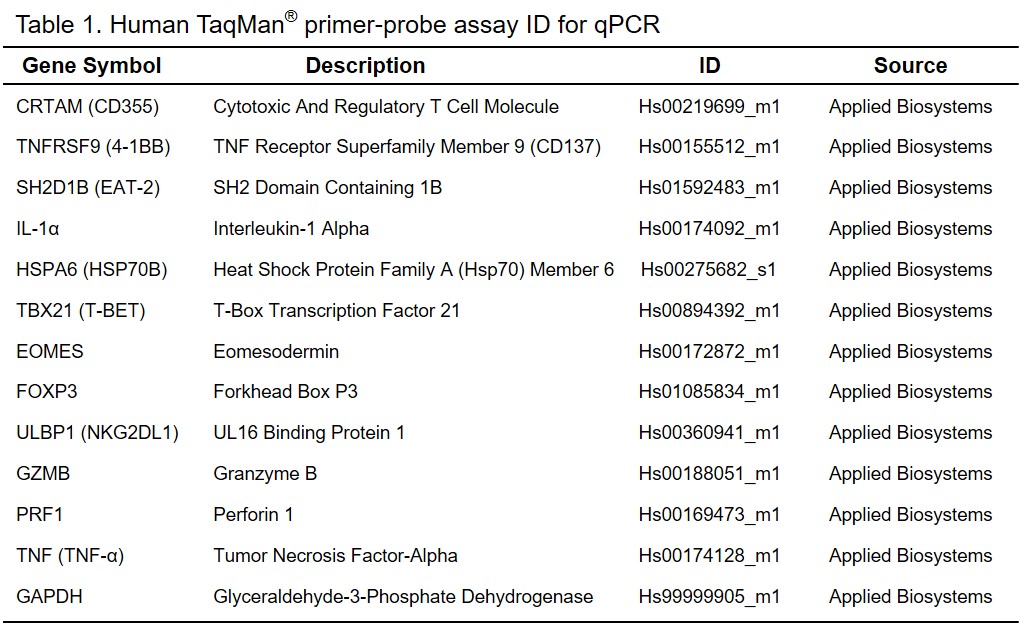


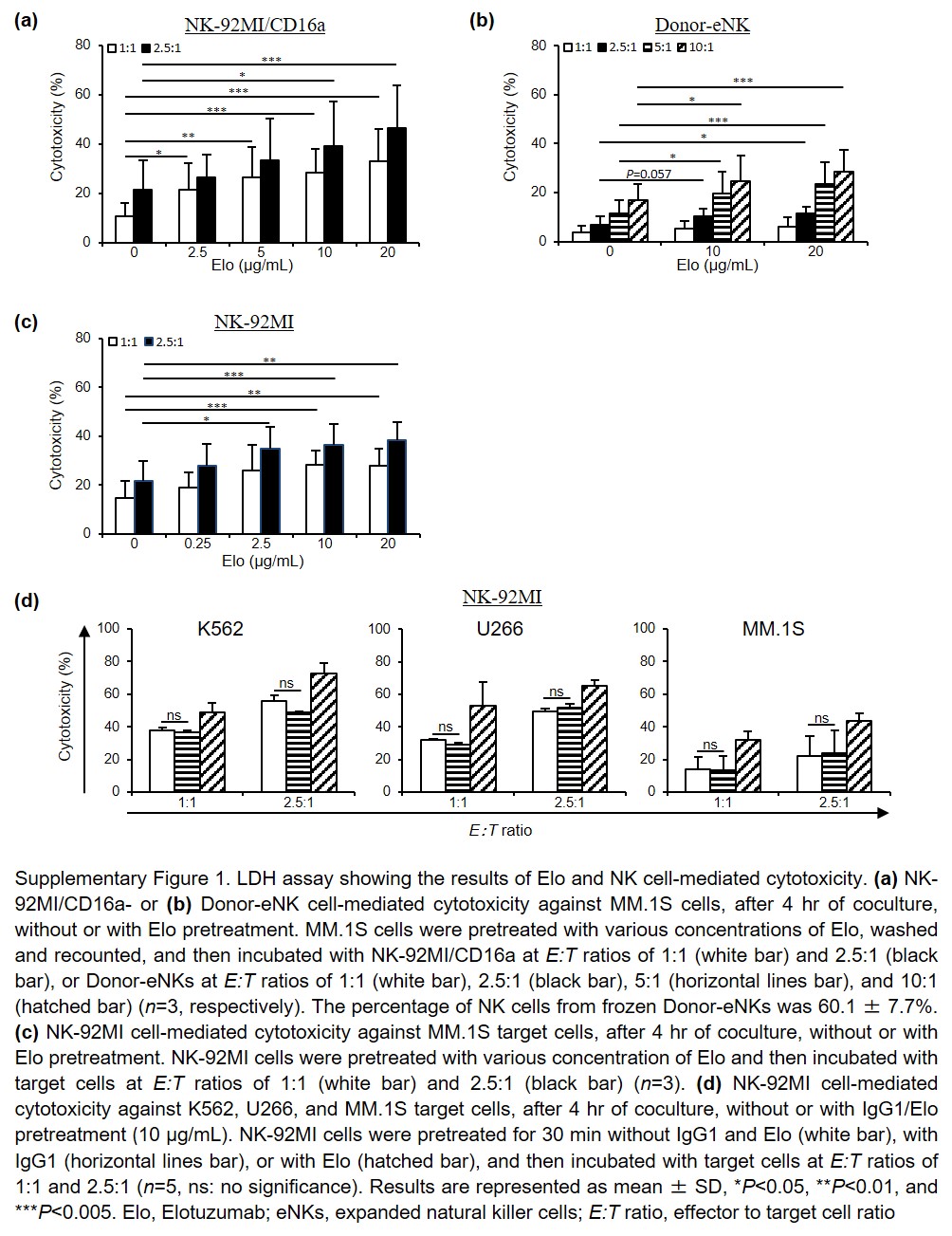


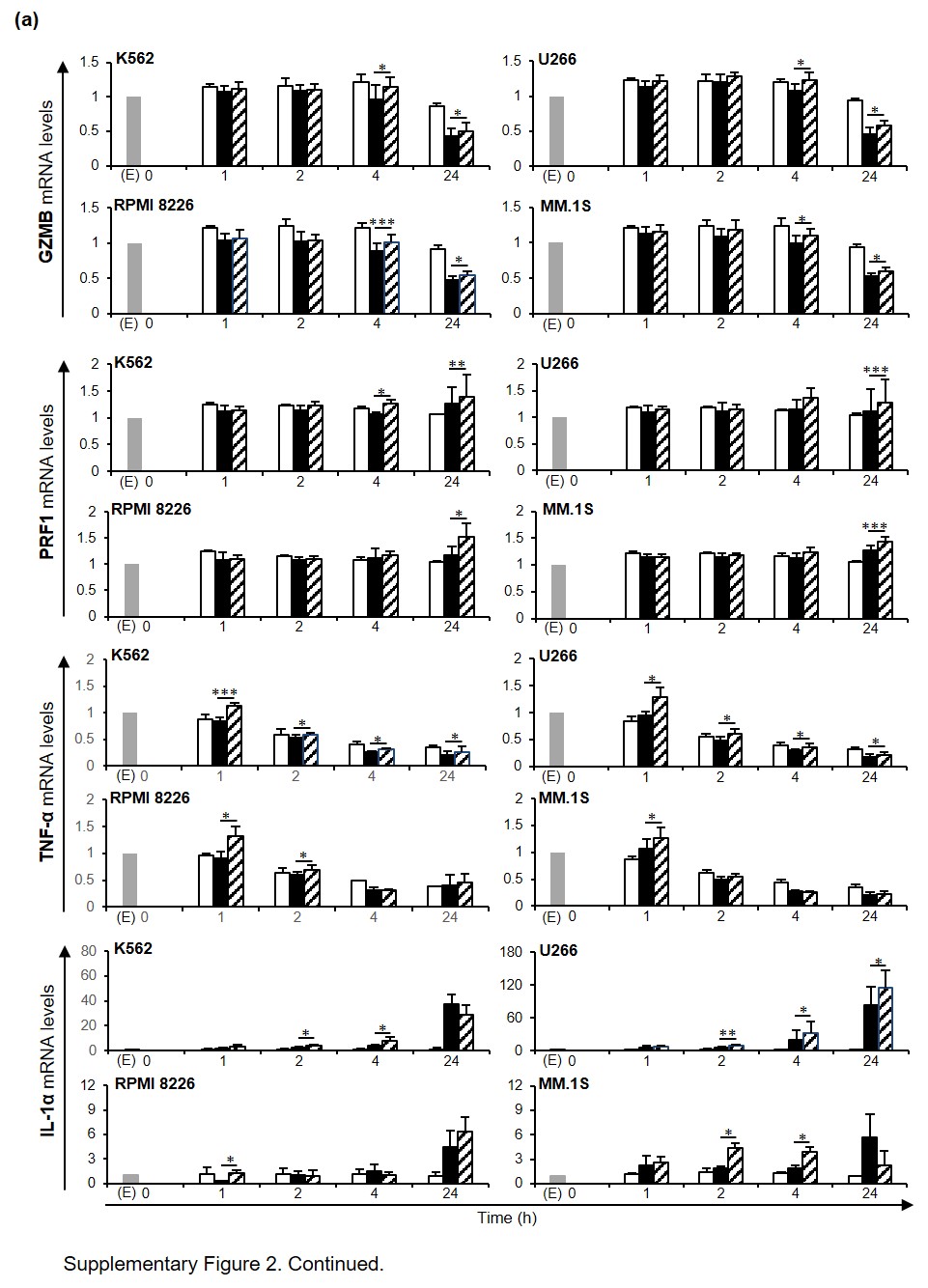


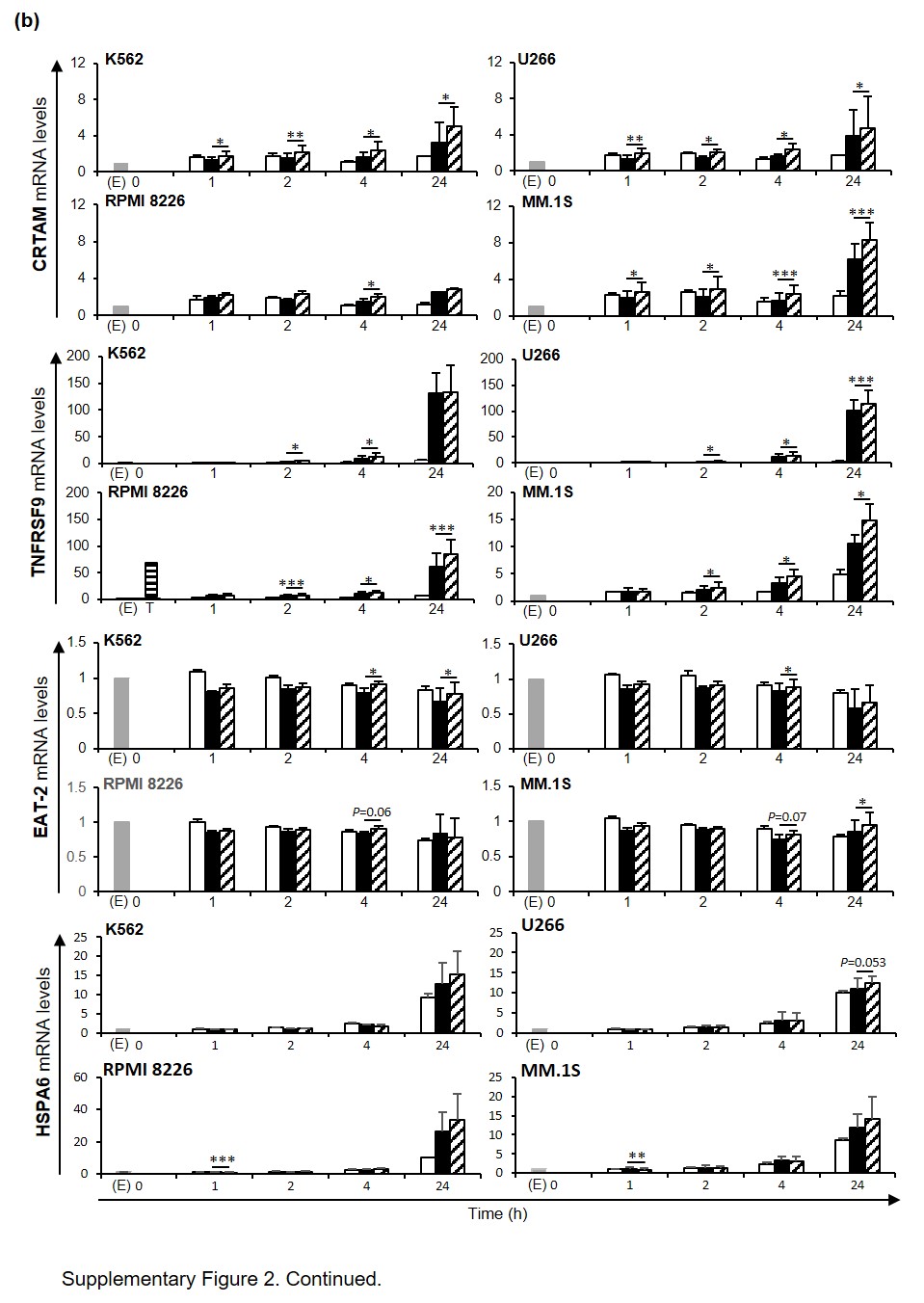


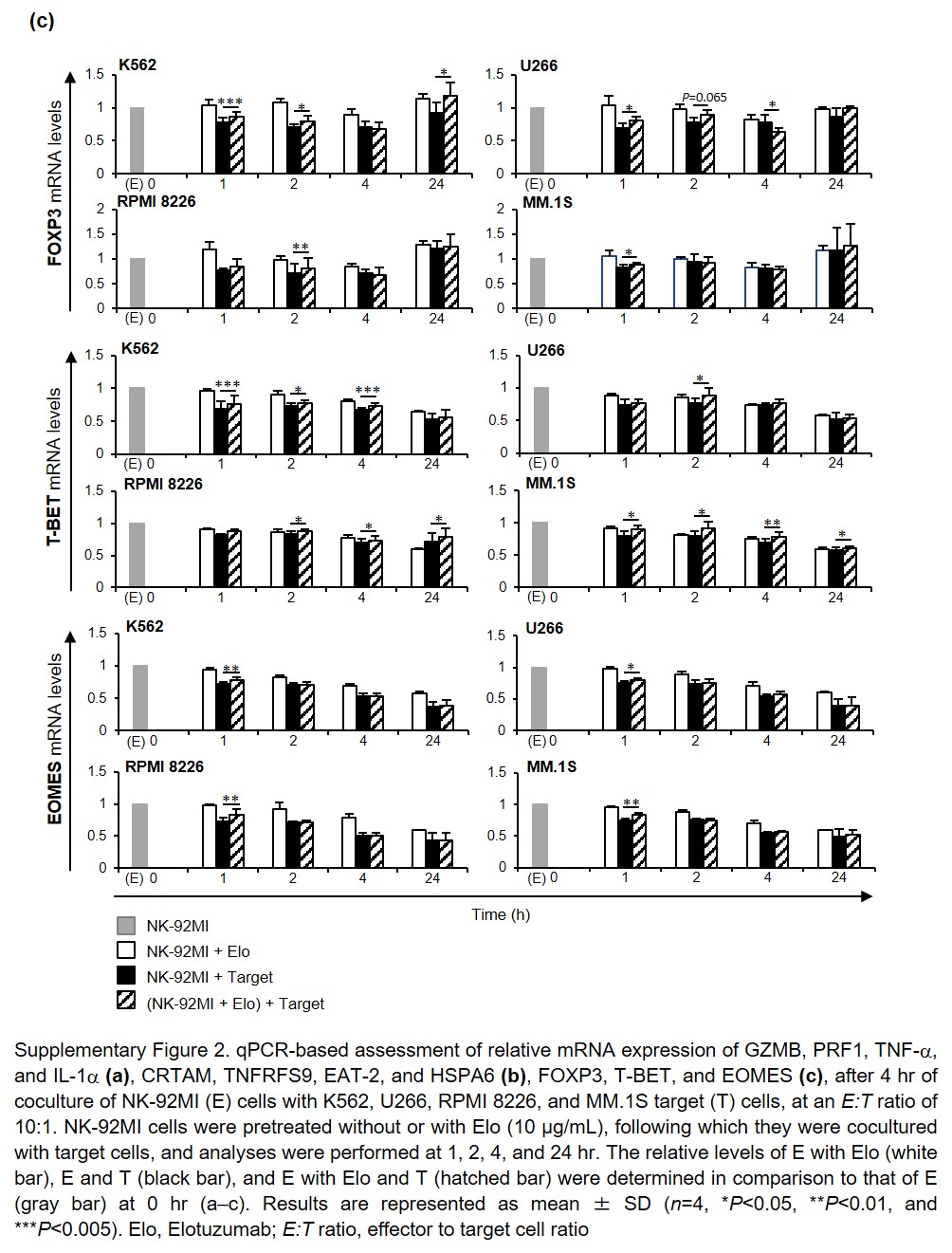


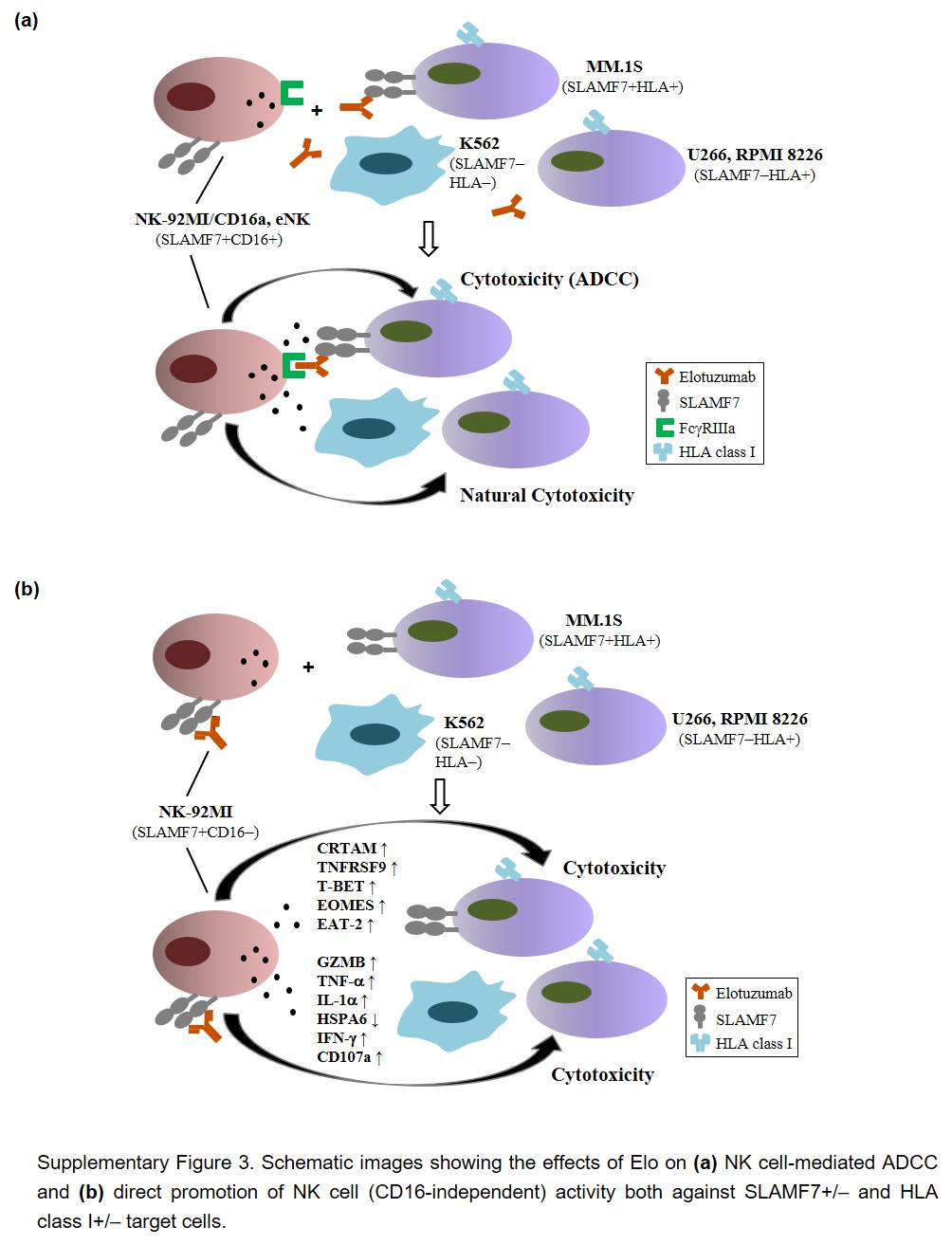

Supplement: Supplementary Materials — Table S1: human TaqMan® primer–probe assay ID for qPCR. Figure S1: LDH assay analysis showing the NK cell-mediated cytotoxicity against target cells pretreated without or with control/different concentrations of Elo antibody. Figure S2: qPCR assay-based assessment of relative mRNA expression of genes after coculture of target cells with NK-92MI cells pretreated without or with Elo, compared to that the expression in NK-92MI cells at 0 hr. Figure S3: schematic images showing the effects of Elo on NK cell-mediated ADCC and direct promotion of NK cell (CD16-independent) activity both against SLAMF7+/– and HLA class I+/– target cells. [file 1429879.f1.docx]
